# Methodological considerations for behavioral studies relying on response time outcomes through online crowdsourcing platforms

**DOI:** 10.1038/s41598-024-58300-7

**Published:** 2024-04-02

**Authors:** Patrick A. McConnell, Christian Finetto, Kirstin-Friederike Heise

**Affiliations:** Integrative Neuromodulation and Recovery (iNR) Laboratory, Department of Health Sciences and Research, Medical University of South Carolina, 77 President Street, Charleston, SC 29425 USA

**Keywords:** Computational biology and bioinformatics, Psychology

## Abstract

This perspective paper explores challenges associated with online crowdsourced data collection, particularly focusing on longitudinal tasks with time-sensitive outcomes like response latencies. Based on our research, we identify two significant sources of bias: technical shortcomings such as low, variable frame rates, and human factors, contributing to high attrition rates. We explored potential solutions to these problems, such as enforcing hardware acceleration and defining study-specific frame rate thresholds, as well as pre-screening participants and monitoring hardware performance and task engagement over each experimental session. With this discussion, we intend to provide recommendations on how to improve the quality and reliability of data collected via online crowdsourced platforms and emphasize the need for researchers to be cognizant of potential pitfalls in online research.

## Introduction

Relying on human intelligence is essential to infer neural mechanisms underlying human behavior. This insight has not changed since the invention of the original Mechanical Turk^1^ and has regained recent attention, for instance, in the field of neuroscience^2,3^. As the Mechanical Turk relied on human chess masters to operate it, so does human behavioral research depend on human participants.

Over the past decade, experimental behavioral research has increasingly leveraged online crowdsourcing platforms for participant recruitment, a trend accelerated with the onset of the COVID-19 pandemic and subsequent closure of physical research facilities^4,5^. Amazon Mechanical Turk (MTurk), originating in 2005 as one of the oldest crowdsourcing platforms, remains widely used in academia as evidenced by its mentions in PubMed-indexed publications rising from under 10 annually in 2013 to around 200 in 2022. This represents a doubling of publications citing MTurk from the pre- to post-pandemic period, illustrating the platform's widespread use across diverse experimental paradigms—ranging from simple surveys to labor-intensive experimental designs like probabilistic learning^6^ and cognitive control task batteries^7^—demonstrating appeal to researchers across many fields^8^.

However, it's imperative to acknowledge the breadth and diversity of the online research ecosystem to contextualize the current state of online data collection more fully. Platforms and panels such as Prolific, Crowdflower (recently rebranded as Figure Eight), CrowdResearch, Qualtrics, and Dynata offer varied participant pools and have been recognized for their robust data quality, catering to a broad spectrum of research needs and disciplines^9,10^. For instance, Prolific has emerged demonstrating superior data quality compared to MTurk and other platforms, particularly in the critical domains of attention, comprehension, dishonesty, reliability, and engagement as assessed via questionnaire^9,11,12^. Utilization of Prolific has increased over the pandemic period similar to MTurk^9^. Importantly, most head-to-head platform evaluation studies assess questionnaire-based data collection and studies demonstrating platform superiority in reaction time-dependent cognitive tasks are scarce^11^; therefore, quality of data is subject to change on all available platforms and there is limited evidence supporting one platform over another in terms of reaction time studies.

Among many experimental paradigms in psychophysics, studying response times constitutes the primary outcome to infer underlying cognitive control processes and assess changes in cognitive functioning^13^. The precise evaluation of response latencies is of particular interest, especially when investigating behavioral change over time, for example, to study learning and memory^14–16^. Unfortunately, several significant methodological shortcomings have been reported^17–20^, which cause considerable obstacles to interpreting reaction time measurements collected online. Additionally, significant time-of-day variations in demographic composition^21^, inadequate worker comprehension of task instructions^22^, worker inattentiveness^23,24^, non-random attrition (i.e., loss of participants over time)^25^, bots, and poor data quality^26^ have been documented. We here add to this list of potential pitfalls with a focus on the reliability of timing-based outcome parameters.

Based on preliminary findings from an ongoing proof-of-concept longitudinal procedural learning study using MTurk, we expand upon the known limitations of online crowdsourcing data and specifically shine a light on the challenges resulting from longitudinal data collection relying on time-sensitive outcomes. Navigating circumstances such as these is of high relevance to research involving psychophysics broadly, and therefore, we discuss two major points, which we consider essential in this context: technical difficulties arising from hardware and software configurations and complications arising from human factors. In discussing the implications of these hurdles, we want to motivate a discussion about potential solutions for improving data quality and provide guidance for researchers planning to use online crowdsourcing platforms to conduct psychophysics research.

## Methods and results

### Overview of the experimental design and data collection process

Response times were collected through an experiment that was created using PsychoJS and then hosted on Pavlovia (https://pavlovia.org/). Participants were recruited via “Human Intelligence Tasks” [HITs] on MTurk (https://www.mturk.com/) and given access to the experiment on Pavlovia. All experimental procedures were conducted in accordance with the ethical principles outlined in the Declaration of Helsinki and approved by the Institutional Review Board for Human Research Committee at the Medical University of South Carolina. The experiment was strictly conducted in accordance with the approved guidelines of the committee.

In the sections below, we introduce related terms such as attrition, drop-out, and noncompliance. To make the relationship between our usage of these terms clear, we operationally define them here as follows: ‘attrition’ is used broadly to encompass the entire scope of participant dropout and data loss that results in an incomplete dataset; ‘drop-out’ is specified as the event when a participant, although initially approved for continuation to the next session, fails to submit it for various reasons or is disqualified based on pre-determined criteria such as insufficient baseline performance or lack of evidence of task learning; ‘noncompliance’ refers to instances where participants do not adhere to task instructions, such as providing incorrect responses during screening, failing to submit tasks within the required timeframe, or demonstrating other behavior that indicates failure to follow instructions—such as entering random key presses or not pressing any keys whatsoever.

To briefly outline the experiment, participants (i.e., workers) went through seven contiguous daily experimental sessions preceded by a screening session (session 0) in which the experiment was described and written informed consent was actively obtained. The experimental paradigm required participants to match visual cues to keys on their keyboard and prompted them to respond with their best possible spatiotemporal precision. Additionally, the screening session was used to filter out bots and inattentive workers based on a simple question querying the number of visual cues corresponding to the keyboard layout—an obvious detail of the informed consent instructions. Critically, sequential information presented during the task spanned two layers: spatial accuracy (i.e., pressing the correct key) and temporal accuracy (i.e., correct timing of on- and offsets of key presses relative to the presentation of the visual cues). The main outcome parameter of the longitudinal experiment was the change in spatiotemporal precision over time, used as an index of skill learning in our experiment. To ensure full functionality of the experimental paradigm, e.g., correct representation of cues, we hardcoded the browser type (i.e., Chrome and Edge) as a requirement to be able to start the experiment.

Participants were informed about requirements for admittance to subsequent sessions and reimbursement upon compliance, defined as adhering to the instructions, and consistency of performance. Noncompliance was explicitly defined as the reason for rejection whereas level of performance was specified as a requirement for qualification (i.e., admittance) to the subsequent session. Workers received payment only for non-rejected sessions. Each of the seven experimental sessions, which were approximately 20–25 min in length, was published on MTurk around noon US Eastern Standard Time [EST]. All sessions were required to be completed within 24 h of publishing to gain access to the respective next session. Access to MTurk HITs was restricted to workers with > 95% prior HIT approval ratings located in countries within Europe and North America at the time of screening. The decision to restrict MTurk HITs to workers in Europe and North America with an approval rating of over 95% follows previous published work within the field of online learning studies, such as those by Bönstrup and others^9,10,16,27^. The restriction to specific regions was informed by the need to manage the HIT postings efficiently, targeting the broad geolocations with one posting per day, which aligned with the demands of our study that include a critical 24-h learning consolidation window. This approach ensures that participants from these regions are likely to be active within the same daily cycle, which is essential for the integrity of our time-sensitive data collection. By delimiting the experiment demographics in this way, via geography and approval rating, we aimed to strike a balance in terms of prioritizing the reliability of collected data and reaching a large, relatively heterogeneous, worker pool. Performance data were manually reviewed daily for task compliance, and submissions were approved and qualified or rejected depending on the criteria mentioned above.

### Point 1: technical factors contributing to attrition

In this section, “technical factors” refers to aspects of an experiment such as user hardware, crowdsourcing platform functionality, interactions between platform (e.g., MTurk) and hosting sites (e.g., Pavlovia), browser settings, and other system-related limitations that might hinder participants' ability to complete tasks as instructed. In the process of developing and refining our experiment, we initially restricted browser access to those based on Google Chrome (e.g., Chromium, Chrome, Edge), but early on after that, we recognized a problem with task performance that required further inquiry into technical factors contributing to attrition. Next, to ensure accurate and reliable response time precision, we closely monitored the frame rates during the study, specifically recording the timing of screen refresh cycles (i.e., the rate at which the screen updates with new visual information) based initially on PsychoJS default calculations and later via custom analysis.

The decision to compute a custom frame rate calculation and closely inspect hardware acceleration arose from our observation of significant variation in frame rate precision with PsychoJS software version updates. Specifically, after updating to a newer version of PsychoJS (i.e., from 2021.2.3 to 2022.2.4), a significant number of sessions with very low frame rates (below 20 frames per second [fps]) were evident. To clarify, the default configuration of PsychoJS, as utilized throughout Pavlovia, performs an initial calculation of the frame rate at the start of the experiment. This initial measurement is a one-time assessment and does not account for variations that may occur as the experiment progresses. Comprehensive documentation on PsychoJS's standard frame rate calculation is available at the core_Window module of PsychoJS, which can be found here: https://psychopy.github.io/psychojs/core_Window.js.html. Based on our observation of frame rate variability, we then implemented two checks in our experimental setup to address the potential absence of hardware acceleration and its impact on frame rate.

First, we initially used PsychoJS’s reporting to verify if the frame rate was above 15 fps—a somewhat arbitrary threshold chosen based on the reasoning that most monitors have a minimum refresh rate of 30 Hz. If the frame rate fell below this threshold, we prompted the user with a warning message. This message advised them to ensure that hardware acceleration was enabled in their browser settings, that the browser was up-to-date, and that no excessive background processes were running on their system that could affect performance.

Second, following discussions with the PsychoJS development team, we implemented their custom version (2022.2.4 custom) of the software that could directly detect and log whether hardware acceleration was enabled. In cases where it was disabled, the software provided a warning message to the user indicating that hardware acceleration was not active. Only through this step, it was possible to identify cases in which graphics processing unit [GPU] based hardware acceleration was disabled, and to automatically switch to an alternative renderer optimized for central processing unit [CPU] rendering.

Thus, while relying on PsychoJS via Pavlovia to calculate frame rates at the beginning of the experiment, we moved to a custom frame rate calculation method that involved averaging the frame times during each experimental trial as well as a custom version of PsychoJS that allowed tracking of hardware acceleration. The advantage of this custom frame rate calculation method was that it allowed the examination of frame rate variability within and across experimental sessions. Our frame rate calculation relies on the fact that the execution of the PsychoJS update functions is locked to the frame rate, or window flip. Throughout the experiment, we use the time stamp at each call of the update function of the routine that runs the trials. The delta change between time stamps of successive trials is used as a measure of the frame time (i.e., effectively frame rate). We then average the frame time for all frames in a trial to calculate the average frame rate for each trial across an experimental session.

This method offers a more granular and continuous assessment of the frame rate throughout the experiment, capturing variability that might be missed by less frequent or static measurements. Our approach ensures that the calculated frame rates are updated and averaged throughout the experiment, providing a more accurate and representative measurement of the participants' actual experience (in our opinion). This custom frame rate calculation method is particularly beneficial in longitudinal studies, where consistent frame rate accuracy is crucial for the reliability of response time measurements indexing learning over time. We here report the custom frame rate calculations only; for completeness, a comparison with the frame rate implemented through Pavlovia is presented in supplemental online information available at 10.6084/m9.figshare.23499099.

Altogether, the addition of these checks, warnings, and custom calculations to our experimental setup was designed to minimize the impact of hardware acceleration issues on the quality of data collected during our study. It is important to note that users do not typically need to explicitly enable hardware acceleration as ‘enabled’ is the default selection for Chrome. Thus, disabled hardware acceleration can be due to several factors beyond the control of our experiment's interface. The most common reasons for hardware acceleration being disabled include (a) the absence of a GPU that supports necessary libraries, such as WebGL, in the user's system, and (b) the user's browser settings might have hardware acceleration disabled, which can happen inadvertently or through manual adjustment for various reasons unrelated to our study. As shown in our results reported below, enabling hardware acceleration is critical for acquiring reliable and accurate response times. In contrast, disabled hardware acceleration results yield untrustworthy data (i.e., low, and variable frame rates), which in our case led to inacceptable data quality and hence discarded crowdsourced data.

**Figure 1:**
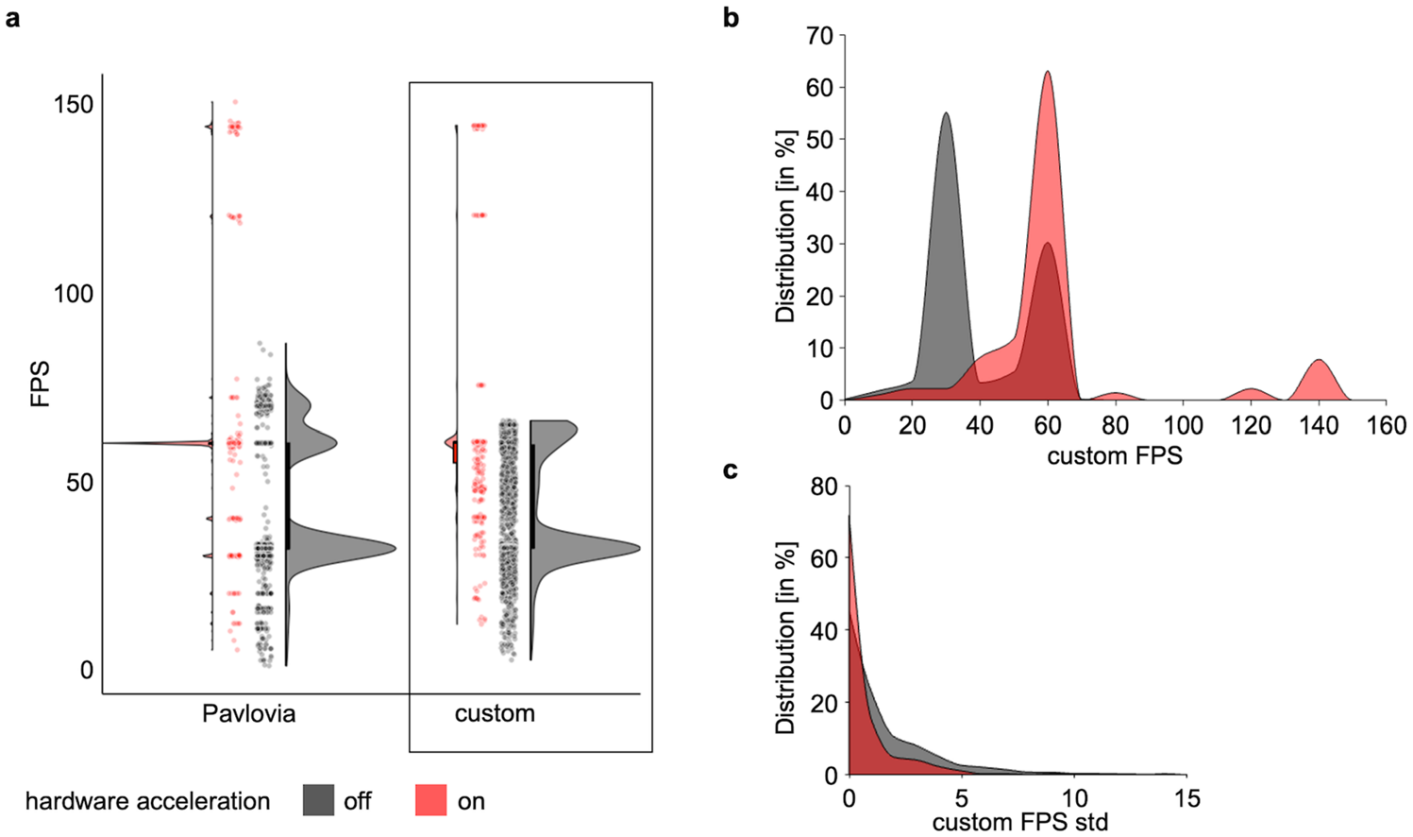
Hardware acceleration influences on frame rate and frame rate variability. Hardware acceleration disabled (‘off’) shown in black, enabled (‘on’) depicted in red. (**a**) Comparison of Pavlovia and custom frame rate calculations. Frames per second (FPS) shown for both frame rate sources, i.e., Pavlovia’s built-in (left) that sampled one data point per session onset, and custom (right), which sampled frame rate across trials within session. (**b**) Normalized distributions of frame rate. Depicts effects of hardware acceleration on custom frame rate calculation across all available data with hardware acceleration ‘on’ showing higher and less variable frame rates than ‘off’. (**c**) Variability distributions of custom frame rate. Depicts within session, between-trial variability (standard deviation, std) of custom frame rate for individual workers with hardware acceleration ‘on’ showing less variability within session than ‘off’. Data and code to reproduce the figures are available at 10.6084/m9.figshare.23499195.

Of a total of 4028 sessions across all 7 experimental days, 119 sessions were run with PsychoJS 2021.2.3, and 3909 were run with PsychoJS 2022.2.4. Hardware acceleration information was available for a subset of these sessions (n = 3781); only 499 (13.20%) were run with hardware acceleration and the vast majority, i.e., 3282 (86.80%), were run with hardware acceleration disabled. Hardware acceleration information was available for 781 unique workers. Of these, 683 workers (87.5%) did not have hardware acceleration enabled, while only 98 (12.5%) did. While the sample running with hardware acceleration also showed lower frame rates in some cases, it was overall considerably more consistent, with most of the data points at 60 fps and a small number at higher frame rates (144 fps) (Fig. 1).

### Point 2: human factors contributing to attrition

As we now turn to explore potential “human factors” contributing to attrition, it becomes evident that while technical issues related to hardware acceleration and frame rate can be identified and rectified with relative ease, human factors introduce a layer of complexity that is less straightforward to navigate. The interaction between technical and human factors, particularly concerning the ignoring of warning messages and task instructions (i.e., noncompliance), remains an outstanding challenge to understand and address.

To identify potential sources of attrition due to human factors, we tracked the number of participants who passed through the various stages of screening and approval, as well as those who failed to submit data or were rejected based on task performance (i.e., drop-out). Additionally, we specifically included text in the reminder emails and in the MTurk HIT material for each session (in large, bright-red bolded lettering), including screening (i.e., “**ATTENTION**: Please **enable** hardware acceleration in browser settings (e.g., Chrome) **before** proceeding to the task. Not enabling hardware acceleration will result in poor frame rate and rejection of submitted HIT!”). These measures were taken to ensure that users had the necessary information to successfully complete the task, although it was not within our capacity to hardcode the hardware acceleration requirement nor to directly modify user system settings or browser configurations.

**Figure 2:**
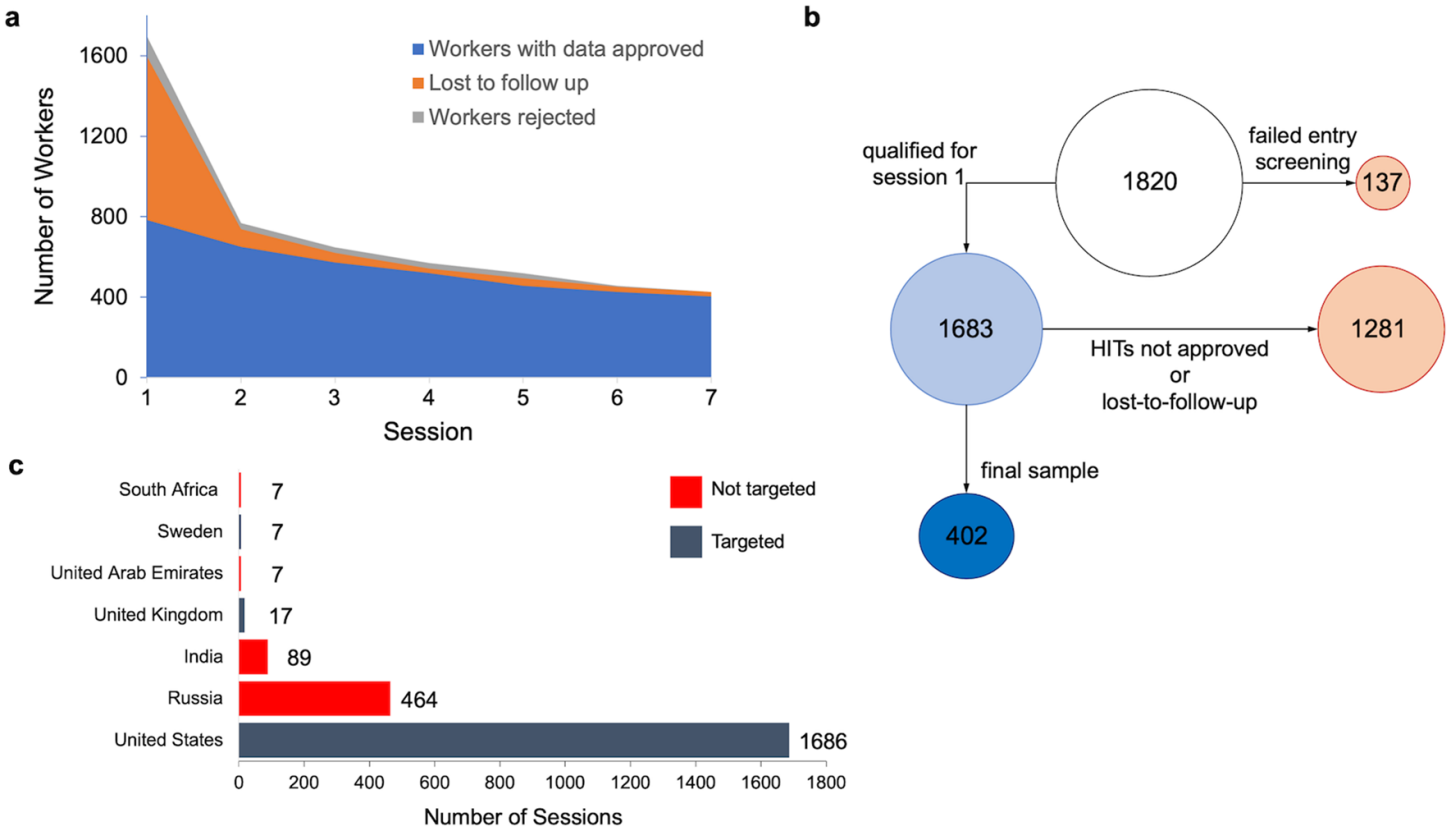
Sources of attrition and noncompliance over time. (**a**) Worker attrition over 7 consecutive days. Colors reflect data loss due to rejection (gray, top), loss to follow-up (orange, middle), and data retained (blue, bottom) over the seven-day experiment. (**b**) Flowchart of attrition from screening to final sample. Diminishing circle sizes reflect the loss of data from screening through session 1 and session 7. Circle colors reflect data loss due to noncompliance (orange, right), data retention (blue, left), and final sample (dark blue, bottom left). Numbers within circles reflect worker count. (**c**) Geolocation of workers across 2277 sessions. Colors represent whether the country from which data originated was on the list targeted for recruitment (dark blue) or not (red). Data and code to reproduce (**a**) and (**c**) are available at 10.6084/m9.figshare.23499198.

Out of the 1820 participants who actively provided informed consent, 137 (7.53%) failed to pass the initial screening question qualifying for admission to the first experimental session. Thus, a total of 1683 (92.47%) workers were eligible to proceed to session 1. The highest drop-out rate, encompassing both participants who did not continue after being approved and provided with the next HIT (n = 798), and those who were “filtered out” based on our predefined criteria (n = 101), occurred during session 1 due to noncompliance with task instructions, resulting in only 784 participants (43.08%) qualifying to move forward to session 2. Our predefined criteria included having sufficiently high frame rates and demonstrating evidence of learning across sessions. This filtering process is akin to the practice in fear conditioning studies where only “responders” are retained for analyses, ensuring that the data reflects genuine learning effects. Across sessions 2–7, further noncompliance (i.e., due to lost-to-follow-up or rejection of the submitted data) led to final approval of only 402 datasets (22.09%) (Fig. 2).

To investigate a possible association between frame rate, lack of hardware acceleration, and geolocation of workers in the sample, we used an application programming interface [API] call to www.ipify.org to record the participants’ internet protocol [IP] address during the experiment, and www.ipapi.com to geolocate the IP addresses during post-processing. Location information was available via IP address for 458 (25.16%) consented and screened participants across a total of 2277 sessions. We did not detect regional differences in hardware acceleration usage contributing to the study findings. Furthermore, the geolocation results indicate that workers submitted MTurk assignments from seven countries, specifically the United States, Russia, India, the United Kingdom, the United Arab Emirates, Sweden, and South Africa.

While most submissions (74.04%) were from the United States, a large proportion of data (24.90%) originated from countries that were not on the approved list for the HIT on MTurk, namely Russia, India, United Arab Emirates, and South Africa (Fig. 2c, above). We recognize the well-known fact that the potential for virtual private network [VPN] usage poses challenges to ensuring the geographical accuracy of the participant pool for online data collection. The unreliability of IP addresses for verifying participants' geographic location has indeed been mentioned as a potential source of concern in the crowdsourcing literature although reports of reported residence to IP address tracing correspondence range from 3 to 10%^25,28,29^. We report that within a sub-sample of our data for which IP address was logged, 24.9% of session data came from untargeted countries of origin—a substantial difference compared to the 3–10% range reported already in the literature. While Amazon Mechanical Turk markets itself as a platform capable of providing a geographically targeted workforce, the reality of its efficacy is less certain.

To help elucidate the distinguishing of technical from human factors, we cite a series of direct communications received from a study participant as in-depth single-case feedback on the experiment. In one case, a sophisticated MTurk user performed the task on a high-end technical setup (i.e., 11th Gen i7 processor with GTX 3060 graphic card and external monitor with 144 Hz refresh rate) and was clocked at 3.129 fps, which corresponded subjectively with task lag and poor perceived performance. Further, the participant noted that upon loading the task (via Google Chrome), they noted a significant increase in local processing demand (“upon running the program, it caused my computer fan to go full blast like a jet engine, which prompted me to open my diagnostic on a separate monitor… I was alarmed to see my laptop temperature consistently stay at 90–95 °C for the full test duration). During the 2nd session, the participant noted a subjective improvement in perceived task performance, and the recorded frame rate had tripled from 3 to 9 fps but remained below our 15-fps criterion for data inclusion. The participant also noted that they indeed saw a warning regarding the low frame rates but was able to just ignore it, and that the warning remained on-screen for 1 s and then disappeared, allowing them to click through since this was not hardcoded as a block to the experiment. On the 3rd session, the participant used Microsoft Edge (which is based on Chrome and indistinguishable from Google Chrome in our diagnostic browser tracking) with a recorded 144 fps and reported a subjective improvement in perceived task performance with no demanding processing load. Additionally, this prompted the participant to check their Chrome settings to discover that the setting for hardware acceleration was in fact turned off. Upon re-enabling hardware acceleration for Google Chrome for session 4, the recorded frame rate was again 144 fps, and no user-end difficulties were noted in the experiment.

In summary, in our longitudinal study, we identified two major sources of data fallibility. On the one hand (technical factors), low and variable frame rates biased data quality by introducing measurement error in assessing reaction time, which could be mitigated through custom PsychoJS code but primarily affected sessions in which hardware acceleration was disabled. On the other hand (human factors), our longitudinal study was affected by high attrition, 77.91%, due to noncompliance and poor data quality leading to the rejection of submissions (n = 221 total rejected HITs) that exceeded the usual loss-to-follow-up (see supplement: 10.6084/m9.figshare.23499198). While geolocation data analysis did not reveal any clear patterns between frame rates and hardware acceleration utilization across regions, we received nearly a quarter of the submitted data from regions outside the locations explicitly specified as inclusion criteria on MTurk and for which we had obtained approval from the local Institutional Review Board.

## Discussion

Online crowdsourcing platforms such as MTurk provide significant advantages over laboratory settings for experimental feasibility, for example, when aiming at larger than typical in-lab-collected sample sizes. Despite these benefits, the experiences gleaned through our experiment confirm known challenges and reveal significant additional ones that need to be carefully considered at all phases of online research. In this report, we identified two main sources of bias in time-sensitive data. The first involves technical shortcomings that affect data sampling, such as low and variable frame rates. The second pertains to human factors, particularly high attrition rates likely amplified, potentially, by psychological factors such as motivation and competing attentional demands. Specifically, Jun et al. reported that they identified five motivation types for participating in online studies: boredom, comparison, fun, science, and self-learning, and found that these motivation types significantly affected study selection, attention, attrition, and study results^30^. These factors compromise the accuracy of response time measurements and can impact accurate inferences. While our reflection is based on our experiences with MTurk, other online platforms (e.g., Prolific Academic [ProA], Crowdflower, Qualtrics, CloudResearch) are likely to face similar liabilities to varying degrees^10,12,31,32^.

While extensive data filtering and rejection are inherent in the design and analysis of online crowdsourced experiments, the extent of data exclusion varies across studies. In our research, stringent criteria based on the precise temporal demands of our task resulted in the exclusion of participant data that did not meet these criteria. This practice aligns with the meticulous approaches adopted by researchers in the field^14,16,33^. Though it is common for studies to report the omission of trial outliers at a rate of approximately 2.5%, we recognize that the complete exclusion of participant data and its impact on the overall dataset is less frequently disclosed in detail^34^. Many studies discuss trial outlier omission and cite excluding entire participants—yet do not transparently report how many or how the exclusion of that data impacts the total amount of excluded data relative to the entire dataset. Our research adheres to a stringent yet necessary data exclusion protocol to ensure the integrity and reliability of our findings. This protocol has led to the exclusion of a significant portion of data when participants' performance did not meet our predefined criteria for learning and frame rate consistency, in line with the precision required for the cognitive processes studied. Our aim in highlighting this key issue—and the implications for experimental design and reporting—is to contribute to the dialogue on research transparency and resource allocation. In the following sections, we discuss our findings in light of their consequences and propose prospective solutions for the design of behavioral online experiments and the reporting of results, as well as technical developments required to ameliorate excessive burden on resources and wasting of collected data.

### Point 1. Technical issues contributing to attrition: hardware acceleration and frame rate

Low and inconsistent frame rates, as demonstrated in our experiment, are major sources of inaccurate response time measurements, both within and across sessions. Taken together, the data show that: (a) frame rate tended to be lower and more variable with hardware acceleration off, (b) variability across sessions was high, regardless of hardware acceleration, and (c) within session, between-trial variability tended to be greater with hardware acceleration off. This compromise on the reliability and validity of study outcomes is primarily attributable to factors like participants' software compatibility and hardware configurations, mainly hardware acceleration. Beyond hardware acceleration, other specific issues known to contribute to low and variable frame rates include the operating system used, with MacOS displaying less temporal precision than Windows or Ubuntu for visual stimuli, and the type of keyboard, as standard USB keyboards can introduce further latencies^18^.

### Techniques to manage frame rate variability

To control these factors, some researchers suggest enforcing GPU hardware acceleration strictly in experiments where single-frame deviations or “dropped frames” are unacceptable^20^. We argue that the effect of disabled hardware acceleration may be even more widespread, and not specifically limited to very high precision latency measurements, in the context of frame rate variability across longitudinal study designs. We implemented custom PsychoJS code as a solution to mitigate frame rate issues without GPU hardware acceleration by optimizing CPU rendering, although it's unclear whether this software solution offers a comparable level of data reliability and consistency. Therefore, we concur with the recommendation to strictly enforce hardware acceleration not only when single-frame deviations are unacceptable but also when substantial variability in frame rate within and across sessions is also intolerable.

Other researchers recommend employing within-subject designs, limiting participants to a single browser, and restricting devices used (e.g., operating system, laptop vs. desktop, USB vs. Bluetooth keyboard, etc.) to reduce experimental noise^17^. Contrary to this recommendation, our data suggest that frame rate variability within and across sessions remains a significant source of bias leading to unreliable data even in within-subject designs. Browser types do represent another significant limitation and cause of variability because it largely remains opaque how they, in general, and with each version update specifically, interact with hardware acceleration status or impact frame rate^18,20^. One approach may be to restrict access to the experiment only through specified browsers, which again is another source of bias and limitation of participant diversity and does not account for unknown interactions between browser version updates and frame rate. Rather than strictly limiting participant hardware and software, implementing software solutions that control or standardize frame rates can help ensure consistent response time measurements within sessions, across sessions, and across participants.

Incorporating robust data screening and cleaning methods early on in a study can help mitigate the impact of frame rate variability detriment on study outcomes, for example, by defining acceptable frame rate thresholds for exclusion prior to beginning data collection. Considering that a standard computer monitor operates at a minimum of 30 fps, it is necessary for researchers to define a frame rate cutoff below this threshold; for example, 20 fps may strike a reasonable balance between hardware limitations and data reliability. Implementing a 20-fps frame rate, latency errors can range from 0 to 50 ms resulting in a potential error of 50 ms, which may be acceptable for some but not all experimental designs. It is important to note that the impact of frame rate on data quality may not be a simple binary issue but requires a more comprehensive exploration into whether there is a continuum of reliability as frame rate increases. Therefore, establishing clear guidelines for acceptable frame rates is essential to ensure the reliability of time-sensitive outcomes. However, researchers need to determine the acceptable margin of error based on their study design, decide whether hardware acceleration is a firm requirement, and then define a threshold of frame rate for exclusion, tailored to each individual study. While limiting study inclusion based on hardware acceleration and frame rate threshold a priori may limit sample diversity and bias results, ignoring these considerations may lead to excessive attrition and data wastage.

### Point 2. Human factors contributing to attrition: task comprehension and compliance

Our findings suggest that attrition rates in online crowdsourced studies can exceed 75% within a one-week timeframe—with the bulk of data loss from screening through session 1—substantially impacting study planning, budget, and time constraints. When screening is effective, however, much data loss can be prevented early on, leaving a remainder of participants committed to the longitudinal design. Besides the logistical implications of such high attrition, the resulting data loss clearly diminishes a study's statistical power and increases the risk of biased results (e.g., selective attrition)^35^. Several factors likely contribute to this concern. First, the lack of face-to-face interaction might result in participants feeling less committed to completing the tasks. Second, typical research designs used to investigate procedural learning with high repetition rates clearly tax motivation and compound loss of participants' interest over time. Third, the remuneration provided might not be perceived as adequate compensation for the time and effort required.

Another related challenge in navigating attrition is noncompliance in task execution. High HIT reject rates can occur due to participants not completing large portions of an experiment during a session or inappropriately performing a task (e.g., random key presses). Noncompliance such as this can largely be rooted out through screening and rejection during the first few sessions but may arise even in later sessions in workers whose performance has met criteria up until that point. In some cases, participants might be multitasking, putting additional load on their system, which leads to inconsistent response times that further contribute to frame rate variability. In these ways, technical and human factors interact in contributing to overall study attrition.

Notably, in the present proof-of-concept experiment reported on here, most workers ignored multiple salient experimental prompts warning them to ensure that hardware acceleration was enabled and that all background processes were turned off prior to beginning each experimental session (at risk of submission rejection) via on-screen prompts and email reminders. Risk of rejection theoretically serves as a potent motivator for MTurk workers to comply with task requirements, although, as shown in our results, it was clearly insufficient in motivating most workers to enable hardware acceleration prior to beginning each experimental session.

### Strategies to reduce attrition due to human factors

One strategy to mitigate attrition and increase retention over several sessions is to use stepwise payment schedules as financial incentives. It is also important to ensure that study designs are engaging and not excessively laborious; this can also help maintain participant interest and commitment, which may, consequently, lead to the decision that a study design is not suited for online data collection.

Pre-screening participants for the likelihood of commitment based on past performance—as it is possible through limiting experimental access based on prior performance scores implemented on online platforms—and ensuring adequate hardware/software capacity are essential steps. This process helps identify the most likely participants to complete the study and provide high-quality data. While these measures may reduce attrition, they will obviously limit the diversity and extent of the worker pool accessed during recruitment.

Ensuring that participants understand the experimental requirements and excluding non-human entities, such as bots, are the two most essential goals of entry screening. Clear communication about study requirements is fundamental to enhancing participant motivation and reducing attrition due to drop-out (i.e., failure to complete and submit HIT on time) or misunderstanding of task instructions (i.e., resulting in poor task performance and exclusion from further HITs). Instructions should be concise and clear, with screening questions implemented to ensure comprehension. It is worth noting that stringent screening and exclusion based on inattentiveness to instructions can reduce sample sizes by over 50% across crowdsourcing platforms^10^. Researchers may benefit from including attention checks during the screening that confirms understanding of hardware acceleration requirements and task comprehension. Alternatively, including a sample task would allow for the determination of hardware acceleration status and task comprehension prior to enrollment in the full study. Post-enrollment, implementing meticulous screening methods, such as daily data reviews based on transparent pre-defined quality criteria, is instrumental in assuring high data quality. As an example, performance metrics can be defined in advance for each experimental session, with stringency increasing as the study progresses.

Ultimately, while the reasons for data exclusion (i.e., HIT rejection) can be made explicit, the human factors contributing to drop-out are largely unknown and commentary can be speculative at best. We recommend that researchers consider including language in their informed consent documentation, and allocate additional budget, allowing for the administration of follow-up surveys to those who drop out. To gain deeper insights into human-related dropouts, we advocate for the implementation of follow-up surveys post-dropout, despite the anticipation of low response rates. Through this probing of human factors contributing to drop-out, we can begin to incorporate such findings to improve online study designs.

In addition to these recommendations, we argue that researchers need to make an informed decision about online crowdsourcing platform usage, such as MTurk, and whether these companies provide reliable and trustworthy services to fulfill requirements for rigorous science. The structure and operations of online platforms directly influence the quality of data collected, and certain improvements are essential to enhance researchers' trust in the data. In our view, the discussed problems we identified with MTurk recruitment underline the need for reform.

Firstly, there is the inability to ensure that workers are meeting geolocation requirements as advertised by MTurk. Despite these claims, our data show that although we specified thirty countries to recruit from through MTurk's service, our geolocation analyses revealed that this filter was not effective in limiting recruitment to the defined regions. Claims regarding the efficacy of geolocation restriction are ineffectual given the ease and accessibility of VPN clients. At best, online platforms can enhance their control by not only restricting access to HITs based on geolocation during screening, but also for each session. While this would minimize variability, it could prevent access to workers who may be traveling between countries over the course of an experiment. The solutions in this regard are unclear. Another potential concern is bias in country representation due to the time of publishing, around noon EST in our case. Even though this timing should not impact which countries are recruited from, it likely influences the geographic distribution of participants and skews the representation of certain regions^21^. Geolocation services need to be reliable, and this discrepancy presents a serious challenge for researchers who need to target specific geographic areas for their studies and may need to exclude data collected from outside the targeted regions.

Secondly, there's the inability to confirm that workers only have one worker ID. We have come across a case where a worker admitted via email that they were enrolled in the study twice with different worker IDs. Although hopefully a rare occurrence, this compromises the validity of study outcomes and further erodes the trust researchers place in a platform. Other related issues concern the possible use of multiple accounts per worker, perhaps facilitated by using different IP addresses via a VPN, or multiple workers sharing the same worker ID. But, as Casey et al. (2017) noted, shared IP addresses do not necessarily indicate the same worker repeating a task^21^. Researchers need to be aware of these limitations and implement their own software solutions to obtain detailed information about their participants, such as browser type and version, hardware specifications, and IP addresses. This information can help researchers identify potential issues that might affect the data quality and increase the burden of attrition and squandered resources.

### General conclusions and recommendations

Here, we report challenges and potential pitfalls intrinsic to online crowdsourced research and discuss measures to mitigate them. Monitoring frame rates and employing suitable software solutions to require hardware acceleration are critical to maintaining the accuracy of response time measurements. Strategies to promote task engagement and limit attrition are also essential, and could include sending email reminders, incorporating frequent attention and comprehension checks, leveraging the stigma of MTurk HIT rejection, and devising a thoughtful payment schedule that motivates participants with fair compensation while also considering the project's budget and realistic attrition rates. Acknowledging the specificity of our context, these conclusions drawn from MTurk data do not generalize to other crowdsourcing platforms per se, due to varying technical setups and participant demographics. Technical issues such as browser compatibility and hardware acceleration, although not unique to MTurk, can impact data differentially depending on the task design and each platform's unique infrastructure. Additionally, our longitudinal approach offered insights into participant engagement that may not translate to studies with different designs such as single-session studies. Crucially, however, the reason behind the extensive disabling of hardware acceleration remains unclear, emphasizing a potential cross-platform challenge that necessitates further investigation. This perspectives report underscores the need for researchers to be cognizant of the potential pitfalls associated with online crowdsourced research and to implement appropriate measures to ensure reliable and valid data collection. By acknowledging and addressing these challenges, researchers can continue to leverage the flexibility and appeal of online crowdsourcing platforms like MTurk, thereby facilitating effective psychophysics research. In conclusion, while there are challenges associated with conducting online crowdsourced research, there are also many potential solutions and areas for future research.

## Data Availability

All source data to reproduce the figures are available under 10.6084/m9.figshare.23499195, 10.6084/m9.figshare.23499198, 10.6084/m9.figshare.23499099.

## Code availability

All code to reproduce the figures is provided alongside with the source data.
